# Coupling imaging mass cytometry with Alcian blue histochemical staining for a single-slide approach

**DOI:** 10.3389/fimmu.2024.1379154

**Published:** 2024-04-29

**Authors:** Patrice Hemon, Danivanh Ben-Guigui, Margaux Geier, Marine Castillon, Corentin Paranthoen, Jacques-Olivier Pers, Marion Le Rochais, Arnaud Uguen

**Affiliations:** ^1^ Lymphocytes B, Autoimmunité et Immunothérapies (LBAI), Univ Brest, Inserm, Centre Hospitalier Universitaire (CHU) de Brest, UMR1227, Brest, France; ^2^ Institute of Oncology and Hematology, Centre Hospitalier Universitaire (CHU) Brest, Brest, France; ^3^ Department of Pathology, Centre Hospitalier Universitaire (CHU) Brest, Brest, France

**Keywords:** imaging mass cytometry, Alcian blue, digital pathology, fixative, histochemical staining

## Abstract

Imaging mass cytometry (IMC) is a metal mass spectrometry-based method allowing highly multiplex immunophenotyping of cells within tissue samples. However, some limitations of IMC are its 1-µm resolution and its time and costs of analysis limiting respectively the detailed histopathological analysis of IMC-produced images and its application to small selected tissue regions of interest (ROI) of one to few square millimeters. Coupling on a single-tissue section, IMC and histopathological analyses could permit a better selection of the ROI for IMC analysis as well as co-analysis of immunophenotyping and histopathological data until the single-cell level. The development of this method is the aim of the present study in which we point to the feasibility of applying the IMC process to tissue sections previously Alcian blue-stained and digitalized before IMC tissue destructive analyses. This method could help to improve the process of IMC in terms of ROI selection, time of analysis, and the confrontation between histopathological and immunophenotypic data of cells.

## 1 Introduction

Deciphering the cell contents, behaviors, and interactions at the microscopic level of normal and pathological tissue remains a key to a better understanding of diseases’ pathophysiology. Being the medical field of pathology, it is also crucial for diagnostic, prognostic, and theranostic purposes as in cancer for example. Pathological tissues are preserved from degradation thanks to a fixation process (mainly formalin-based one) before a process leading to paraffin embedding that permits, on the one hand, cutting the tissue thinly (some micrometers of thickness) for staining applications before microscopic analysis and, on the other hand, storing the formalin-fixed paraffin-embedded (FFPE) samples for years for potential complementary analyses conditioning the patient’s treatment given the advances in medical applications and/or for research applications (1).

Beyond analyzing finely the morphology of cells within the tissue, pathologists also routinely used immunostaining methods to point and quantify some protein markers not accessible through the sole examination of a histochemically stained tissue slide. Routine immunostaining methods, based on the revelation of protein-targeting specific antibodies using chromogens (immunohistochemistry IHC) or fluorochromes (immunofluorescence IF), are well-integrated methods in the workflow of pathologists and are more and more used for several of the above-evocated diagnostic, prognostic, and theranostic applications (2, 3). Nevertheless, they only permit the simultaneous revelation of a low number of targets per tissue section (most of the time one or two) preventing any deeper phenotyping of cells and analyses of interactions between finely phenotyped cells in their tissue environment.

To increase the number of markers co-analyzable in single tissue sections beyond the limitations of IHC and IF, imaging mass cytometry (IMC) has been developed as a method permitting to co-analyze approximately 40 differently labeled markers in a single tissue section. Indeed, instead of chromogens or fluorochromes (whose number of non-overlapping channels limits the number of co-analyzable markers using IHC and IF), for IMC, target-specific antibodies are coupled to metal isotopes, each with its proper mass and related time of flight when detected using mass spectrometry. The pool of metal-coupled antibodies is incubated on the tissue section for the fixation of the antibodies to their protein targets, and then a laser ablates each squared micrometer of the tissue for ionization and analysis of its metal content (correlated with the amounts of protein targets within the ablated tissue) thanks to mass spectrometry. By the repetition of this process of laser ablation, tissue ionization, and detection of metal content for each consecutive pixel of a tissue area, IMC builds a data matrix associating for the coordinates of each 1-µm² tissue area to its content in approximately 40 markers corresponding also to a multichannel and 1-µm²-resolution microscopic image of the tissue (4).

Nevertheless, IMC presents some specific limitations. Some of them are related to the preanalytical process of the sample to analyze first, notably the fixation step, which is a well-known factor influencing the ability to reveal protein antigens using IHC but which remains poorly to not studied in the field of IMC (5–7). Novel potential issues specifically point to the IMC process too (and not taken into account for IHC and IF applications) as the potential content in metals within the tissue itself (e.g., from patients treated using platinum-based chemotherapies) or in the solutions used for tissue pre-IMC processing (e.g., different fixatives) (8). In addition, IMC has some intrinsic technical limitations such as the limited resolution of the analysis (1 µm, which did not reach the precision of pathology morphological, IHC, and IF analyses) and the time (and costs) of an IMC analysis: the analysis of successive pixels linked to the laser ablation 1 µm² per 1 µm² requires approximately 100 min to analyze a small tissue area of 1 mm² with a cost related to metal-coupled antibodies and time of acquisition of several hundreds to thousands of dollars/euros per slides (9). Variability within the same section can arise due to technical factors inherent to the IMC process, including variations in tissue preparation, staining efficiency, and imaging conditions. These factors may introduce inconsistencies in signal intensity and spatial resolution, impacting the accuracy and reproducibility of the results. Furthermore, biological heterogeneity within tissue sections presents another significant challenge in IMC analysis. Variability in cellular composition, antigen expression levels, and tissue architecture can influence the interpretation of IMC data and may complicate the identification of meaningful biological patterns. Given these limitations, IMC analyses are often focused on small regions of interest (ROI) instead of large tissue areas.

To optimize the preanalytic process of tissue samples and sections dedicated to IMC analysis including the selection of the ROI, we led a technical study 1) searching for potential different fixative-related factors interfering with IMC analysis and 2) searching for a pre-IMC staining of the tissue that would permit to finely determine the ROI before performing the IMC analysis on the same stained-tissue section.

## 2 Materials and equipment

### 2.1 Tissue selection, fixation, and section

All tissue samples used for this technical study were included in a registered tissue collection of the Department of Pathology of CHU Brest (Brest, France), and the present study was conducted in compliance with the Helsinki Declaration and after approval by our institutional review board (CHRU Brest, CPP no. AC-2019-3642). For one tissue (#1, appendix), only an FFPE (4% buffered formalin) sample was available. For three tissues (#2 colon, #3 stomach, #4 small intestine), different samples were performed from the fresh specimen and fixed using different fixatives: 4% buffered formalin (F4%), zinc formalin (ZnF), acetic acid formalin (AF), and alcohol–acetic acid–formalin (AFA); one additional sample per specimen was 4% buffered formalin-fixed and treated using formic acid mimicking a decalcification process (F4%decal) before paraffin embedding. For subsequent stainings (histochemical, IHC, and IMC), 5-μm tissue sections of the FFPE samples were mounted on Superfrost^®^ Plus slides (Thermo Scientific, Saint-Herblain, France) and dried overnight at 37°C before processing.

### 2.2 Histochemical stains

Hematoxylin–eosin Saffron (HES), Masson’s trichromic stain (MT), Toluidine Blue (TB), Alcian blue (AB), periodic acid–Schiff (PAS), or Giemsa staining procedures were conducted using the automated protocols used daily for diagnostic purposes in the Department of Pathology at CHRU Brest (Brest, France). The histochemical stains were performed using Sakura Tissue-Tek automation and validated staining protocols and products according to the manufacturer’s instructions (Sakura Finetek Europe, Alphen aan den Rijn, The Netherlands).

### 2.3 Immunohistochemistry

Antibodies targeting the following proteins were used for IHC analyses on unstained slides of the differently fixed samples: CD3 (polyclonal antibody, Dako, Glostrup, Denmark, 1:100 dilution), CD20 (clone L26, Dako, 1:100), CD4 (clone SP35, Roche Diagnostics, Meylan, France, pre-diluted), CD8 (clone C8/144B, Dako, 1:25), CD68 (clone PG-M1, Dako, 1:100), EMA (clone E29, Dako, 1:100), Ki-67 (clone MIB-1, Dako, 1:50), smooth muscle actin (clone 1A4, Roche Diagnostics, pre-diluted), pan-keratin (clone AE1/AE3, Dako, 1:200 dilution), CDX-2 (clone EPR2764Y, Thermo Scientific, 1:50), and CD31 (clone JC70A, Dako, 1:50). IHC was performed on a Ventana Benchmark Ultra^®^ automated slide preparation system (Roche Diagnostics) using the ultraView Universal DAB Detection Kit (Roche Diagnostics) and the ultraView Universal Alkaline Phosphatase Red Detection Kit (Roche Diagnostics, for CD20 revelation). For each marker, the slides underwent a pretreatment with cell conditioner 1 (pH 8) for 30 min, followed by incubation with the antibody at 37°C for 20 min. After washing, the slides underwent counterstaining with one drop of hematoxylin for 12 min and one drop of bluing reagent for 4 min. Subsequently, slides were removed from the immunostainer, washed in water with dishwashing detergent, and mounted. For each slide, the staining was quoted by a pathologist (AU) in terms of intensity of the expected label (from 0, no staining, to 3 strong staining) and in terms of unspecific background (from none 0 to 3 strong unspecific staining).

### 2.4 Brightfield slide digitalization

Histochemically stained slides were scanned using a 3DHISTECH PANNORAMIC Midi slide scanner (3DHISTECH, Budapest, Hungary). CaseViewer software (3DHISTECH) was used for the selection and annotations of regions of interest (ROI) in digital slides. The corresponding ROIs were drawn on the verso side of the stained slides before IMC analyses.

### 2.5 Imaging mass cytometry acquisition

IMC analyses were conducted with the Hyperion^®^ Imaging System technology (Standard BioTools) on tissue sections previously incubated (case #1) or not incubated (#2,#3,#4) with a pool of metal-coupled antibodies. Briefly, after antibody incubation and washing, the slide is placed in the Hyperion^®^ Imaging System, where a laser peels off the slide tissue per 1-µm² area. The metallic ions from the antibodies attached to the tissue are differentiated in the function of their time of flight (ToF). For each 1 µm² of analyzed tissue, the automated device determines its content in the various metal ions. The data produced are generated in the form of.mcd and.txt files exploitable for cellular, phenotypic, or neighborhood analyses.

In non-immunolabeled histochemically stained slides of tissue #1, small areas of 10 µm × 10 µm were used to evaluate the mean counts on 135 mass channels with assignments of different laser energies (−10 dB, −5 dB, 0 dB, and +5 dB).

With the non-immunolabeled slides of tissues #2, #3, and #4, tissue ROIs were extended to 100 µm × 100 µm with a laser energy set to +5 dB. Iridium-stained slides (iridium being used for cell nuclei counterstaining in the IMC process) were used as controls and as the reference for slide comparisons. An average count inferior to 1,000 was necessary to avoid the risk of degradation of the detector, with the risk of degradation becoming medium to high with more than 1,000 and more than 10,000 counts, respectively, according to the manufacturer’s instructions. These measures were performed searching for metal contained in the tissue sections that could interfere with the IMC process itself and the detection of metal-coupled antibody-related signals.

Tissue slides (without previous histochemical stain for one slide and with our different histochemical stains for the others) were incubated with 24 metal-coupled antibodies. Immunolabeled slides of tissue were then acquired using the Hyperion in a 1-mm² ROI per slide searching for the impact of histochemical stains on the level of fixation of the different antibodies in comparison with a non-histochemically stained slide. The following markers and corresponding metals were used: CD45 (89Y), CD38 (141Pr), STIM1 (142Nd), vimentin (143Nd), CD14 (144Nd), Tbet (145Nd), Pan-keratin (148 Nd), CD11b (149Sm), CD31 (151Eu), CD45(152Sm), CD11c (154Sm), FoxP3 (155Gd), CD4 (156Gd), CD68 (159Tb), CD20 (161Dy), CD8a(162Dy), CD127 (168Er), collagen type I (169Tm), CD3 (170Er), CD27 (171Yb), caspase-3 cleaved (172Yb), CD45Ro (173Yb), HLA-DR (174Yb), pS6 (175Lu).

## 3 Methods

### 3.1 Objective and validation of the method

We propose an optimal strategy 1) using neutral formalin 4% fixative for the fixation of tissue and 2) using an Alcian blue staining and slide digitalization for the determination of the ROI before the IMC process himself and data acquisition.

### 3.2 Step-by-step procedure

#### 3.2.1 Day 1

1. Fix the tissue with 4% buffered formalin (F4%) and fixation duration time depending on tissue nature and size.

#### 3.2.2 Day 2

2. Cut 5-μm tissue sections of the FFPE sample.
3. Put the tissue section on slides with a special treatment process that electrostatically adheres to tissue sections.
4. Dry tissue sections overnight at 37°C.

#### 3.2.3 Day 3

5. Deparaffinize tissue section in xylene and rehydrate through graded ethanols to deionized water.
6. Stain in the Alcian blue solution for 30 min.
7. Rinse in a wash solution for 5 min.
8. Counterstain in nuclear fast red for 5 min.
9. Rinse, dehydrate, and clear the slide with a film coverslip.
10. Scan the AB-stained slide using a scanner.
11. Select and annotate regions of interest (ROI) in digital slides using the viewer software associated.
12. Draw the corresponding ROI on the verso side of the stained slide before IMC analyses.
13. Take off the coverslip.
14. Perform antigen retrieval of the tissue section with a Tris-EDTA buffer (cell conditioner 1 pH 8) for 30 min.
15. Cover the tissue with 100 μL of primary metal-tagged antibodies overnight at 4°C.
16. Fast wash with ultrapure water.
17. Dry the tissue section for 20 min at 40°C.
18. Place the slide in the Hyperion^®^ Imaging System.
19. Spot the ROI before laser ablation.

## 4 Results

### 4.1 HES and MT stains could cause the degradation of the mass spectrometry detector

**Figure 1:**
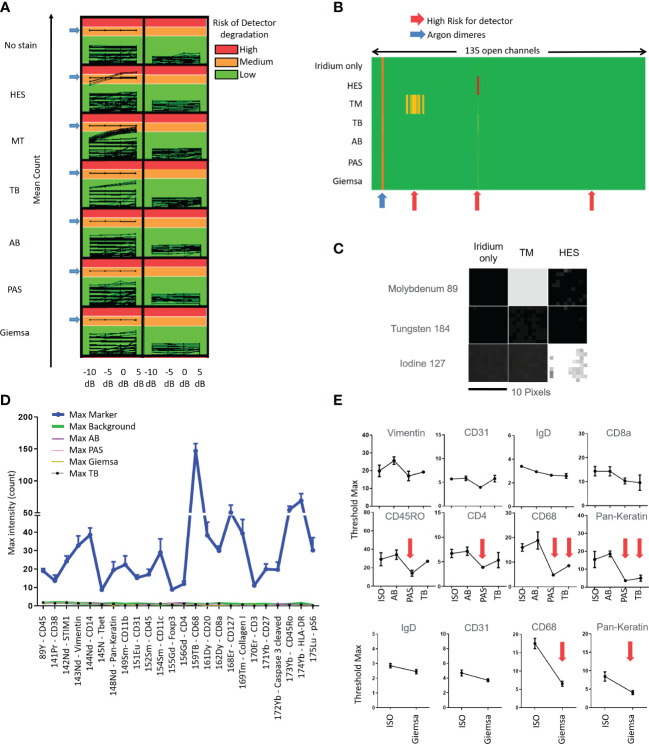
Tests quantifying the impact of slide staining before imaging mass cytometry Hyperion ^®^ data acquisition in terms of risk of degradation of the metal’s detection **(A-C)**, of non-specific noise **(D)** and diminished intensities of immunolabeling **(E)** using the different stains (hematoxylin eosin Saffron (HES), Masson’s trichrome (MT), toluidine blue (TB), Alcian blue (AB), periodic acid–Schiff (PAS), Giemsa; ISO: immunostained only) **(A)** Evaluation of mean count acquired on all 135 channels with increasing laser energies (−10, −5, 0, + 5 dB) of each histological staining. Control is tissue with iridium staining only. An average of low signal intensity for the channels of interest (<1,000 counts, Green) be fine for acquisition in terms of instrumentation. If the average signal intensity for channels is high (>10,000 counts, Red), this high signal could contribute to more rapid detector aging. An average of low signal intensity for the channels between 1,000 and 10,000 counts could contribute to detector deterioration. **(B)** Heatmap representation of the average of mean counts acquired for a laser energy of +5 dB, showing that HES and MT stains could contribute to more rapid detector aging (red arrows). **(C)** Image representation of channels that could contribute to more rapid detector aging, molybdenum (Mo) 92, 94, 95, 96, 97, 98, and 100 for MT and iodine (I) 127 for HES. The acquisition has been done by square of 10 pixels × 10 pixels. **(D)** Histogram representation of the Maximal (Max) intensity counts acquired for the channels of interest. The “Max Markers” correspond to the maximal intensities of markers usually used for each channel and obtained with the positive (i.e., immunostained only) control (antibodies used are indicated in the left table). The “Max type of histological staining” corresponds to the maximum intensities of contamination of each staining in the different channels. Slide control without staining (“Max Background”) was used for control. The mean of max intensity for each histological staining is comparable with the background from no stain (i.e., no histochemical stain and no immunolabeling) slide (red arrow) **(E)** Examples of maximal threshold measurements with several immunomarkers and with unstained slide (no stain) and different stains. No significant diminution was observed using AB stain (significant diminutions are indicated with red arrows).

Applying IMC acquisition to slides of tissue #1 stained with HES, MT, TB, AB, PAS, and Giemsa, we observed that HES and MT stains were demonstrated to accelerate the aging of the metal detector because of ion counts superior to 1,000 in molybdenum (Mo) 92, 94, 95, 96, 97, 98, and 100 channels for MT and 127 iodine channels for HES. On the contrary, no risk of detector degradation appeared with TB, AB, PAS, and Giemsa stains (**Figures 1A–C**). These results were confirmed by performing the same analyses on other F4% fixed samples from tissues #2, #3, and #4. As a result, because they were judged not appropriate to perform IMC analyses, HES and MT stains were excluded from further testing due to concerns regarding potential degradation of the Hyperion Imaging System automation (**Figure 1A**).

### 4.2 The different fixatives are not at risk of detector degradation but could modulate the reactivity of antibodies

Applying IMC acquisition to slides of tissues #2, #3, and #4 1) from samples with different fixation processes consisting of either F4%, ZnF, AF, AFA, or F4%decal and 2) from samples stained with TB, AB, PAS, and Giemsa, we observed no risk of degradation of the detector (i.e., ion counts inferior to 1,000 in every channel) with each fixative process and the four different stains.

Analyzing the IHC results obtained within these samples, we observed no major differences in the staining intensities in terms of expected or artifactual staining except a diminished intensity of specific nuclear staining and an increase of unspecific background with CDX-2 and Ki-67 markers in F4%decal samples.

In this manner, given that the fixation process itself did not prevent performing IMC analyses and did not impair the staining with the different antibodies tested in our study in addition to the case of F4%decal samples, we chose to carry on our technical study by using F4% fixed samples as recommended in diagnostic pathology guidelines.

### 4.3 AB stained tissue sections are usable for IMC analysis

**Figure 2:**
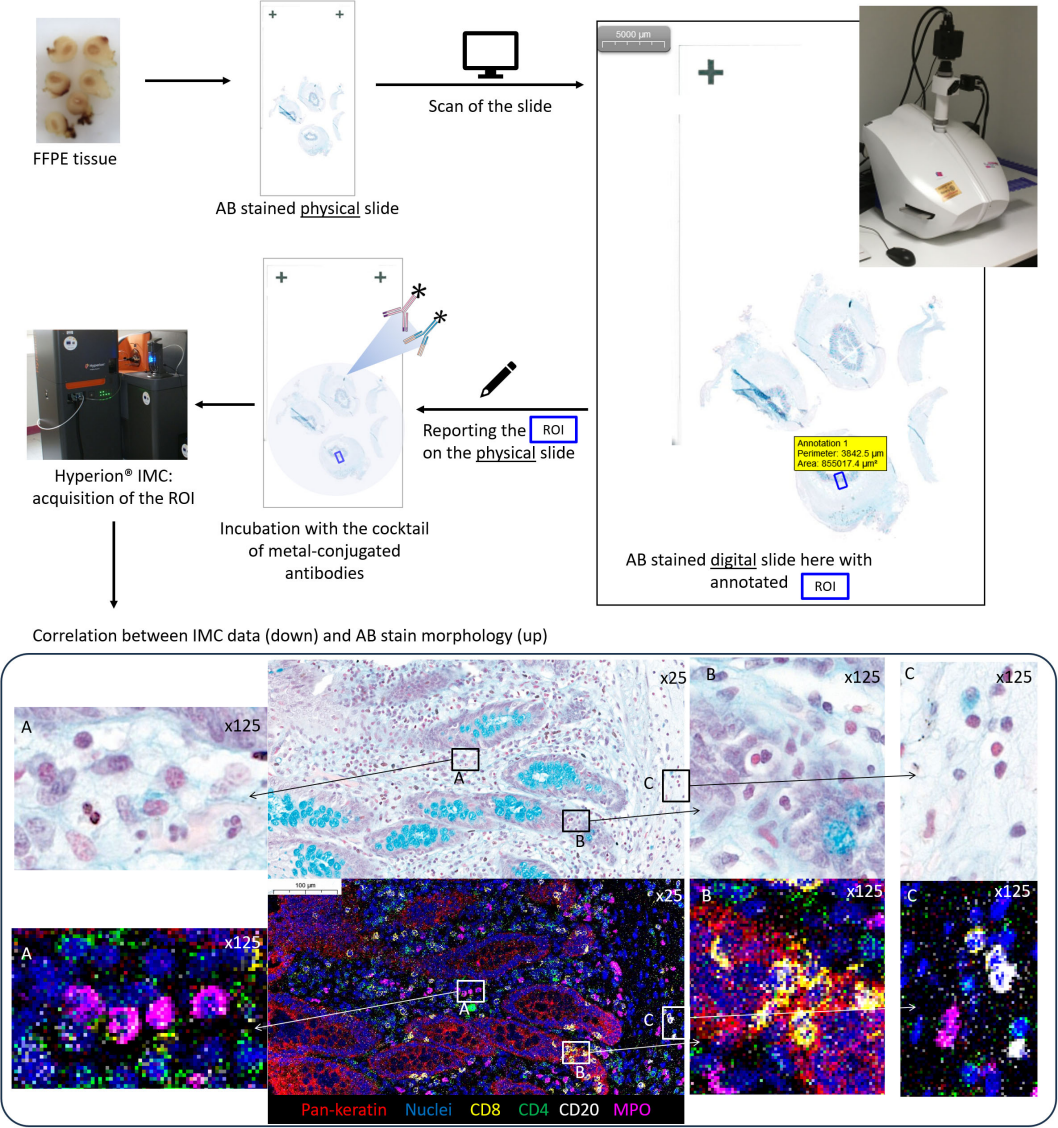
Summary of an analytic pipeline allowing the fine comparison of histochemical stain morphology and imaging mass cytometry (IMC) results within a single tissue section: example of an appendiceal sample. 1: The tissue section is produced based on a neutral formalin-fixed paraffin-embedded tissue sample and Alcian blue (AB) stained; 2: the AB-stained slide is digitalized; 3: the digital AB slide is used for the selection of the region of interest (ROI) for IMC analysis based on microscopic examination; 4: the ROI is positioned on the verso side of the AB-stained slide, which is used for IMC processing; 5: IMC acquisition of the ROI is performed; 6: the co-analysis of IMC data and digital AB slide allows the confrontation of morphological and immunophenotypic features until the single-cell level on the two images: note the exact overlapping and immunophenotyping between single cells within the Alcian blue (AB, CaseViewer, 3DHistech)) stain and IMC data (MCD Viewer, Standard BioTools) within the region of interest (ROI). (A) four neutrophile granulocytes with myeloperoxidase MPO positivity; (B) cytotoxic T cells CD8-positive within the appendiceal epithelium pan-keratin-positive; (C) three B cells CD20-positive and one neutrophile granulocyte MPO positive).

Applying IMC acquisition to slides of tissue #1, we compared the signal intensities for the 24 different markers/antibodies incubated on tissue sections without staining before the immunolabeling or stained with TB, AB, PAS, or Giemsa stains before the immunolabeling step. We observed that the immunostaining intensities decreased with some markers (e.g., pan-keratin, CD68, CD4, myeloperoxidase) in slides previously stained with TB, PAS, or Giemsa whereas only little to no decrease in staining was observed in the slide previously stained using AB (**Figures 1D, E**). In this manner, an AB-stained tissue section of an F4% FFPE sample appeared the most appropriate condition to select the ROI meriting IMC analysis. In addition, beyond the analysis of the AB-stained section using a microscope to select the ROI, the digitalization of the AB-stained tissue before the IMC process permits not only the visualization and selection of the ROI but also the saving of the detailed microscopic morphological features of the tissue before laser ablation for future paired analyses and morphological validation of IMC data. **Figure 2** summarizes our final proposal for an analytic pipeline allowing the fine confrontation of AB-based histopathological image and imaging mass cytometry (IMC) results of a single tissue section.

## 5 Discussion

IMC permits phenotype cells within their tissue context at a higher level than IHC or IF methods. As a method based on the immunostaining of protein targets within the tissue, IMC must be performed on samples responding to the same quality criteria as those recommended for IHC and IF analyses. Nevertheless, due to its specific revelation system based on the detection of metal isotopes using mass spectrometry, it requires specific quality parameters to avoid not only interferences with analytic results but also damages to the IMC automaton, which remains highly costly. In our study, we have demonstrated that different tissue fixation processes used in pathology are also applicable to perform IMC analyses with no risk of detector degradation. In this manner, IMC could be used in a vast spectrum of new and archived pathology samples that could consist of a major source of data of medical and scientific interest.

Beyond its high multiplexing capacities, some limitations of IMC in terms of morphological resolution as well as time and cost of tissue data acquisition could also be diminished thanks to routine pathology processes. For example, in our study, we have shown that it is feasible to perform an IMC analysis on a tissue slide previously stained with different histochemical stains routinely used in pathology laboratories. Because it does not damage the detector of the IMC automaton and does not impair the immunolabeling of cells and tissue, as well as permits the analysis of the microscopic features of the cells and tissue, AB staining is particularly interesting as a “pre-IMC” staining.

At a time when IMC is fast growing in research applications, we believe that coupling IMC and digital pathology will allow gaining information in pairing with high-precision detailed immunophenotypic and morphological features of cells within normal and pathological tissues, leading to a better understanding of physiological and pathophysiological processes. Coupling on a single slide, IMC and digital pathology but also, soon, other innovative tissue-based -omics methods (e.g., proteomics and spatial transcriptomics) will permit further gain in the exploration of healthy and pathological tissue for better disease understanding and innovative treatment proposals.

## Data availability statement

The raw data supporting the conclusions of this article will be made available by the authors, without undue reservation.

## Ethics statement

The studies involving humans were approved by CHRU Brest, CPP n° AC-2019-3642. The studies were conducted in accordance with the local legislation and institutional requirements. The human samples used in this study were acquired from a by-product of routine care or industry. Written informed consent for participation was not required from the participants or the participants’ legal guardians/next of kin in accordance with the national legislation and institutional requirements.

## Author contributions

ML: Data curation, Investigation, Methodology, Project administration, Writing – original draft, Writing – review & editing. PH: Conceptualization, Data curation, Formal analysis, Investigation, Methodology, Project administration, Software, Supervision, Validation, Writing – original draft, Writing – review & editing. DB-G: Data curation, Investigation, Methodology, Writing – review & editing. MG: Data curation, Writing – review & editing. MC: Data curation, Formal analysis, Writing – review & editing. CP: Data curation, Formal analysis, Methodology, Writing – review & editing. J-OP: Conceptualization, Funding acquisition, Resources, Visualization, Writing – review & editing. AU: Conceptualization, Formal analysis, Investigation, Methodology, Project administration, Software, Supervision, Validation, Visualization, Writing – original draft, Writing – review & editing.
